# Synthesis, Single Crystal X-ray Analysis, and Antifungal Profiling of Certain New Oximino Ethers Bearing Imidazole Nuclei

**DOI:** 10.3390/molecules22111895

**Published:** 2017-11-03

**Authors:** Reem I. Al-Wabli, Alwah R. Al-Ghamdi, Hazem A. Ghabbour, Mohamed H. Al-Agamy, Mohamed I. Attia

**Affiliations:** 1 Department of Pharmaceutical Chemistry, College of Pharmacy, King Saud University, P.O. Box 2457, Riyadh 11451, Saudi Arabia; toto24se@hotmail.com (A.R.A.-G.); ghabbourh@yahoo.com (H.A.G.); 2 Department of Medicinal Chemistry, Faculty of Pharmacy, Mansoura University, Mansoura 35516, Egypt; 3 Department of Pharmaceutics, College of Pharmacy, King Saud University, P.O. Box 2457, Riyadh 11451, Saudi Arabia; malagamy@ksu.edu.sa; 4 Microbiology and Immunology Department, Faculty of Pharmacy, Al-Azhar University, Cairo 11884, Egypt; 5 Medicinal and Pharmaceutical Chemistry Department, Pharmaceutical and Drug Industries Research Division, National Research Centre (ID: 60014618), El Bohooth Street, Dokki, Giza 12622, Egypt

**Keywords:** imidazole, Mannich reaction, X-ray, antifungal agents, anti-*Candida*

## Abstract

Fungal infections threaten human health, particularly in immune-compromised patients worldwide. Although there are a large number of antifungal agents available, the desired clinical attributes for the treatment of fungal infections have not yet been achieved. Azoles are the mainstay class of the clinically used antifungal agents. In the current study, the synthesis, spectroscopic characterization, and antifungal activity of certain new oximino ethers **Va**–**n** bearing imidazole nuclei are reported. The (*E*)-configuration of the imine double bond of the synthesized compounds **Va**–**n** has been confirmed via single crystal X-ray analysis of compound **Vi** as a representative example of this class of compounds. The molecular structure of compound **Vi** was crystallized in the monoclinic, *P*2_1_/*c*, *a* = 18.7879(14) Å, *b =* 5.8944(4) Å, *c* = 16.7621(12) Å, *β* = 93.063(3)°, *V* = 1855.5(2) Å^3^, *Z* = 4. The in vitro antifungal activity of the synthesized compounds **Va**–**n** were evaluated using diameter of the inhibition zone (DIZ) and minimum inhibitory concentration (MIC) assays against different fungal strains. Compound **Ve** manifested anti-*Candida albicans* activity with an MIC value of 0.050 µmol/mL, being almost equipotent with the reference antifungal drug fluconazole (FLC),while compounds **Vi** and **Vn** are the most active congeners against *Candida parapsilosis*, being equipotent and about twenty-three times more potent than FLC with an MIC value of 0.002 µmol/mL. The results of the current report might support the development of new potent and safer antifungal azoles.

## 1. Introduction

Fungal infections are an ever-growing burden on the health of mankind. They sometimes cause significant morbidity and mortality particularly in immune-compromised individuals, like those taking anticancer chemotherapy, patients with AIDS, or those receiving organ transplants [1,2]. Invasive fungal infections, like invasive aspergillosis and candidiasis, threaten the human health of millions of patients annually worldwide [3]. The available antifungal drugs can be classified into five main categories according to their mode of action: antimetabolites (e.g., 5-fluorocytosine) [4], polyenes (e.g., nystatin and amphotericin B) [5], azoles (e.g., itraconazole, voriconazole and fluconazole) [5], allylamines (e.g., naftifine and terbinafine) [6], and echinocandins (e.g., micafungin and caspofungin) [7].

Azoles bearing either imidazole or triazole moiety as a pharmacophoric portion constitute the mainstay antifungal therapy due to their good safety profile and favorable bioavailability [6]. They target lanosterol 14α-demethylase (CYP51) enzyme, a member of the CYP51 class of cytochrome P450 enzymes, leading to inhibition of the biosynthesis of ergosterol and accumulation of the toxic methylated sterol, which ultimately results in fungi cell death [8]. Even though azoles are currently the most clinically prescribed antifungal agents, they suffer from some limitations. Inhibition of cytochrome P450 enzymes by azoles leads to interference with the metabolism of other co-administered medications [9]. Moreover, azoles lack fungicidal activity against many fungi, which leads to the development of resistance toward fungal therapy [3]. Therefore, there is considerable interest in developing new azole-bearing antifungal agents endowed with a wide antifungal spectrum, high potency, diminished undesired drug–drug interactions, and reduced adverse effects.

**Figure 1:**
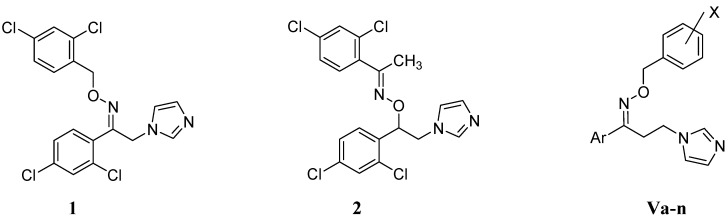
Chemical structures of the antifungal agents **1** and **2** as well as the target compounds **Va**–**n**.

Screening the literature revealed that oxiconazole (**1**, Figure 1) and its inverted oxime analog **2** (Figure 1) are well known antifungal agents bearing both imidazole and oxime functionalities [10,11]. Moreover, most of the currently available azole-bearing antifungal agents feature a spacer of two carbon atoms between the azole pharmacophore moiety and an aromatic nucleus, while insufficient information is available about azole antifungals bearing a three-carbon bridge connecting azole and aromatic moieties [12,13,14]. Accordingly, it was of our interest to synthesize the oximino ethers **Va**–**g** to be evaluated as new antifungal agents bearing oxime and imidazole fragments with a three-carbon atom bridge between the imidazole pharmacophore and the aromatic moiety. In addition, a 1,3-benzodioxole scaffold was incorporated into a plethora of bioactive molecules including antimicrobials [15,16,17]. Therefore, the phenyl ring in compounds **Va**–**g** was replaced by 1,3-benzodioxole moiety to afford the respective compounds **Vh**–**n** to be assessed as new antifungal candidates. Moreover, the configuration around the imine double bond of the title compounds **Va**–**n** was explored via single crystal X-ray analysis of compound **Vi** as a representative example of this type of compounds.

## 2. Results and Discussion

### 2.1. Chemistry

**Scheme 1:**
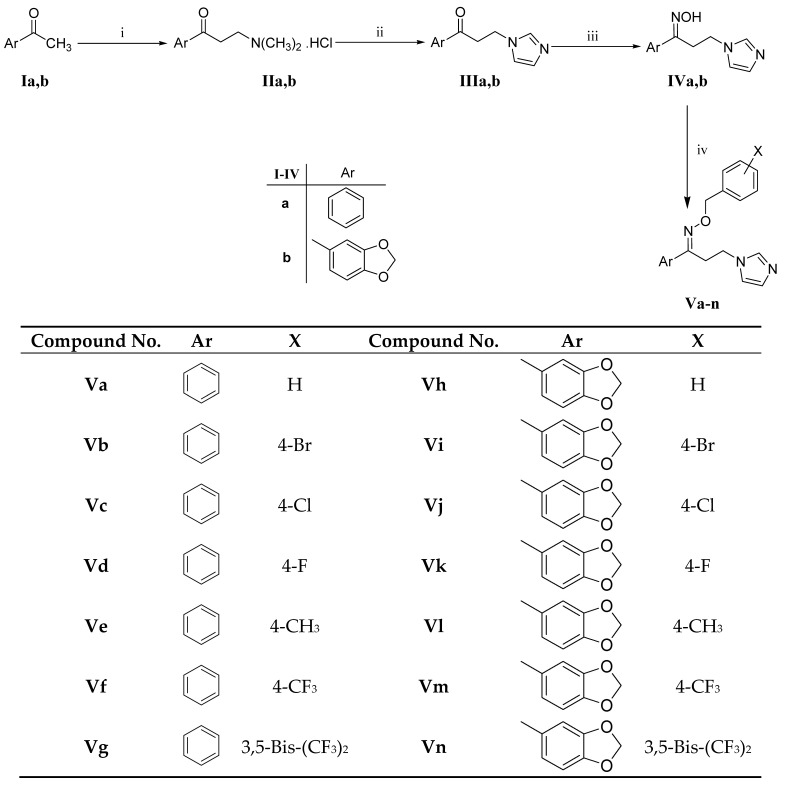
Synthesis of the target compounds **Va**–**n**. Reagents and conditions: (i) HN(CH_3_)_2_.HCl, (CH_2_O)*_n_*, conc. HCl, ethanol, reflux, 2 h; (ii) Imidazole, water, reflux, 5 h; (iii) H_2_NOH.HCl, KOH, ethanol, reflux, 18 h; (iv) Appropriate benzyl chloride/bromide derivative, NaH, DMF, 80 °C, 3 h.

The target compounds **Va**–**n** and their intermediates were successfully achieved as illustrated in Scheme 1. The synthesis was commenced by utilizing the commercially available acetophenones **Ia**,**b** to perform Mannich reactions to give the respective Mannich bases **IIa**,**b**. Compounds **IIa**,**b** were elaborated to the corresponding ketones **IIIa**,**b** which were subsequently transformed to the oximes **IVa**,**b**. The target oximino ethers **Va**–**n** were obtained in 24–63.4% yields via etherification of the pivotal oximes **IVa**,**b** using the appropriate benzyl bromide/chloride in the presence of sodium hydride. The assigned chemical structures of the title compounds **Va**–**n** were confirmed via different spectroscopic techniques (IR, ^1^H-NMR, ^13^C-NMR and mass spectrometry). The aromatic protons of compounds **Va**–**n** appeared along with imidazole protons in the range of 6.75–8.39 ppm, while the benzylic protons occurred in the range of 5.13–5.29 ppm. The benzodiaxole methylene protons of compounds **Vh**–**n** manifested around 5.9 ppm. The aliphatic ethylene protons of compounds **Va**–**n** were observed in the expected upfield region of 3.14–4.29 ppm. The ^13^C spectra of compounds **Va**–**n** showed aromatic as well as imidazole carbons in the range of 106.2–162.6 ppm. Their benzylic carbons occurred at 74.8–76.7 ppm, while the benzoldiaxole methylene carbons of compounds **Vh**–**n** were observed around 101 ppm. Moreover, the aliphatic ethylene carbons of compounds **Va**–**n** exhibited signals in the expected region of 28.9–44.3 ppm while the oximino carbons manifested signals around 155 ppm. The mass spectral data of the target compounds **Va**–**n** are consistent with their assigned chemical structures. The (*E*)-configuration of the oximino double bond of the title compounds **Va**–**n** has been proved using single crystal X-ray analysis of compound **Vi** as a representative example of the prepared compounds **Va**–**n**.

### 2.2. Crystal Structure of Compound ***Vi***

**Figure 2:**
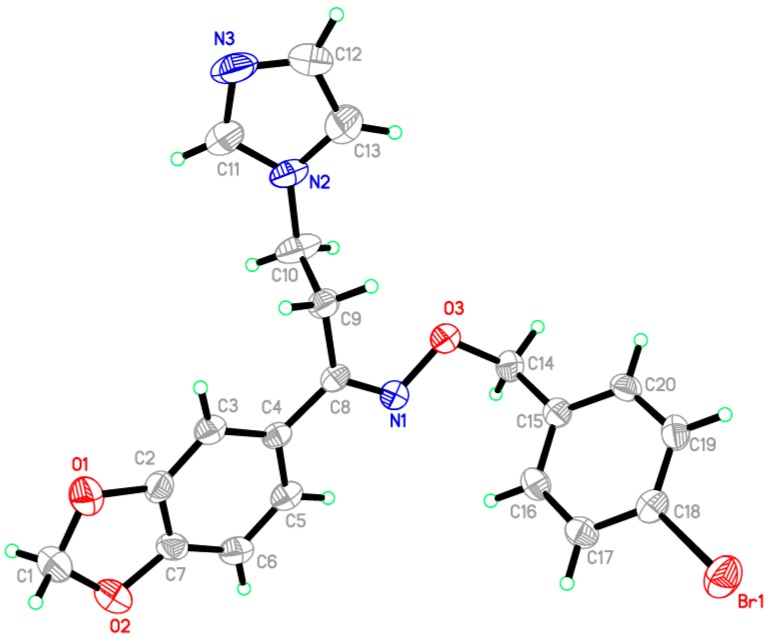
ORTEP diagram of compound **Vi**. Displacement ellipsoids are plotted at the 40% probability level for non-H atoms.

**Figure 3:**
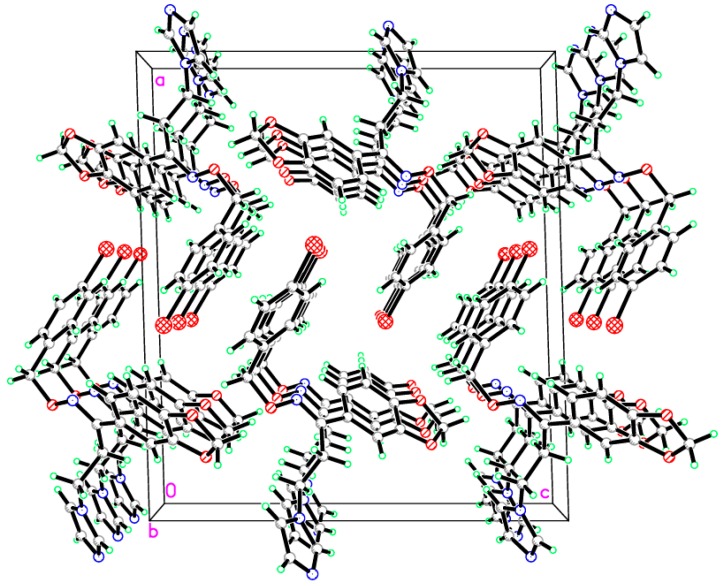
Molecular packing of compound **Vi**.

The selected bond lengths and bond angles of compound **Vi** are listed in Table 1. The asymmetric unit contains one independent molecule as shown in Figure 2. All the bond lengths and angles are within normal ranges [18]. In the crystal packing, Figure 3, molecules are linked via one intermolecular hydrogen bond (Table 2).

### 2.3. Antifungal Evaluation

The antifungal activity of the synthesized compounds **Va**–**n** was determined against three *Candida* species and *Asperagillus niger* using in vitro diameter of the inhibition zone (DIZ) and minimum inhibitory concentration (MIC) assays; the results are presented in Table 3.

### 2.4. Structure–Activity Relationships

The current study reports the antifungal potential of certain imidazole-bearing compounds having either an unsubstituted phenyl ring (compounds **Va**–**g**) or a benzodioxole fragment (compounds **Vh**–**n**) representing the aromatic pharmacophore moieties. The title compounds **Va**–**n** feature 3-aryl-3-iminopropyl moiety attached at *N*^1^ of the imidazole ring. It has been previously reported that the presence of chlorine atoms in the aromatic moiety of the antifungal agents contributes to the enhancement of their antifungal activity [19]. Substitution of the phenyl moiety of the benzyl fragment with halogen, methyl, or 3,5-bis-trifluoromethyl groups of compounds **Va**–**g** enhanced their antifungal activity against the tested *Candida* species. Compound **Ve** bearing 4-methylbenzyl moiety exhibited the best MIC value of 0.050 µmol/mL, being nearly equipotent with the reference fluconazole (FLC) towards *Candida albicans*, while compounds **Vb** (4-bromobenzyl derivative) and **Vg** (3,5-bis-trifluoromethyl benzyl derivative) manifested the best MIC values of 0.083 and 0.36 µmol/mL towards *Candida tropicalis* and *Candida parapsilosis*, respectively. The same anti-*Candida* profile was mostly observed in the respective analogs **Vh**–**n** except towards *Candida parapsilosis* in which substitution with halogen or methyl did not improve the activity as compared with the unsubstituted analog, compound **Vh**. Substitution with the trifluoromethyl group gave compound **Vm** improved activity towards *Candida albicans* and *Candida tropicalis* with, an MIC value of 0.153 µmol/mL. The highest sensitivity of *Asperagillus niger* was observed towards the 4-chlorobenzyl derivative analog, compound **Vc**, with an MIC value of 0.047 µmol/mL. In summary, compounds **Vb**, **Vc**, **Ve** and **Vi**, or **Vn** are the most active congeners towards *Candida tropicalis*, *Asperagillus niger*, *Candida albicans*, and *Candida parapsilosis*, respectively. It seems that the antifungal profile of compounds **Va**–**g** is better than that of their respective benzodioxole analogs, compounds **Vh**–**n**. Therefore, it is believed that the replacement of an unsubstituted phenyl pharmacophore with benzodioxole moiety is not favorable towards the tested fungal strains.

## 3. Experimental

### 3.1. General

The melting points were measured using a Gallenkamp melting point device and are uncorrected. The NMR samples of the synthesized compounds **Va**–**n** were dissolved in DMSO-*d*_6_ and the NMR spectra were recorded using a Bruker NMR spectrometer (Bruker, Reinstetten, Germany) at 500 MHz for ^1^H and 125.76 MHz for ^13^C at the Research Center, College of Pharmacy, King Saud University, Saudi Arabia. Chemical shifts are expressed in δ-values (ppm) relative to TMS as an internal standard. Elemental analyses were carried out at Microanalysis Laboratory, Cairo University, Cairo, Egypt and the results agreed favorably with the proposed structures within ±0.4% of the theoretical values. Mass spectra were recorded using Agilent Quadrupole 6120 LC/MS with ESI (Electrospray ionization) source (Agilent Technologies, Palo Alto, CA, USA).

### 3.2. Chemistry

#### 3.2.1. Synthesis of (1*E*)-1-(2*H*-1,3-benzodioxol-5-yl)-*N*-hydroxy-3-(1*H*-imidazol-1-yl)propan-1-imine (**IV**)

Compound **IV** and its intermediates were prepared as previously reported [20,21]. Their spectral data are consistent with the reported ones.

#### 3.2.2. Synthesis of the Oximino Ethers **Va**–**n**

Sodium hydride (1.5 mmol) was added to a solution of the oxime (**IV**, 1.0 mmol) in DMF (5 mL) and the reaction mixture was stirred at room temperature for 10 min. Then, the appropriate benzyl bromide/chloride (1.1 mmol) in DMF (5 mL) was added dropwise. The reaction mixture was stirred at room temperature for 30 min. then heated at 80 °C for two hours. The reaction mixture was concentrated under vacuum and the residue was poured into ice cold water and extracted with ethyl acetate (3 × 20 mL). The organic phases were combined and washed with water (2 × 15 mL), dried (Na_2_SO_4_), and evaporated under reduced pressure. The crude oximino ethers **Va**–**n** were purified using column chromatography, and chloroform/methanol (18:1) was used as the solvent system.

*(1E)-N-(Benzyloxy)-3-(1H-imidazol-1-yl)-1-phenylpropan-1-imine* (**Va**). Yield 52.9%; light brown viscous oil; IR (KBr): ν (cm^−1^) 3003, 2964, 1724 1673 (C=N), 1506, 1437, 1286, 700; ^1^H-NMR (CDCl_3_): δ (ppm) 3.16 (br. s., 2H, –C**H_2_**–CH_2_–N), 4.12 (br. s., 2H, –CH_2_–C**H_2_**–N), 5.25 (s, 2H, –C*H_2_*–C_6_H_5_), 6.77 (s, 1H, –N–C**H**=CH–N=), 6.94 (s, 1H, –N–CH=C**H**–N=), 7.27–7.31 (m, 5H, Ar–H), 7.33–7.38 (m, 5H, Ar–H), 7.41 (s, 1H, –N–CH=N–); ^13^C-NMR (CDCl_3_): δ (ppm) 29.4 (–**C**H_2_–CH_2_–N), 43.4 (–CH_2_–**C**H_2_–N), 76.7 (–**C**H_2_–C_6_H_4_), 118.8 (–N–**C**H=CH–N=), 125.9, 128.1, 128.3, 128.4, 128.5, 128.6, 129.1, 129.5, 134.8 (Ar–CH, Ar–C, –N–CH=**C**H–N=), 137.3 (–N–CH=N–), 154.9 (C=N); MS *m*/*z* (ESI): 306.2 [M + H]^+^, 307.1 [(M + 1) + H]^+^.

*(1E)-N-[(4-Bromobenzyl)oxy]-3-(1H-imidazol-1-yl)-1-phenylpropan-1-imine* (**Vb**). Yield 43.4%; pale yellow viscous oil; IR (KBr): ν (cm^−1^) 3003, 2924, 1720, 1672 (C=N), 1510, 1436, 1220, 785; ^1^H-NMR (CDCl_3_): δ (ppm) 3.34 (br. s., 2H, –C**H_2_**–CH_2_–N), 4.29 (br. s., 2H, –CH_2_–C**H_2_**–N), 5.24 (s, 2H, –C**H_2_**–C_6_H_4_), 6.94 (s, 1H, –N–C**H**=CH–N=), 7.11 (s, 1H, –N–CH=C**H**–N=), 7.37 (d, *J* = 7.5 Hz, 2H, Ar–H), 7.47–7.57 (m, 5H, Ar–H), 7.62 (d, *J* = 7.4 Hz, 2H, Ar–H), 7.66 (s, 1H, –N–CH=N–); ^13^C-NMR (CDCl_3_): δ (ppm) 29.2 (–**C**H_2_–CH_2_–N), 43.7 (–CH_2_–**C**H_2_–N), 75.8 (–**C**H_2_–C_6_H_4_), 119.0 (–N–**C**H=CH–N=), 122.1, 126.1, 127.7, 128.5, 128.7, 129.7, 130.1, 131.6, 134.5 (Ar–CH, Ar–C, –N–CH=**C**H–N=), 136.4 (–N–CH=N–), 155.0 (C=N); MS *m*/*z* (ESI): 384.1 [M + H]^+^, 386.1 [(M + 2) + H]^+^, 387.1 [(M + 3) + H]^+^.

*(1E)-N-[(4-Chlorobenzyl)oxy]-3-(1H-imidazol-1-yl)-1-phenylpropan-1-imine* (**Vc**). Yield 63.4%; light brown viscous oil; IR (KBr): ν (cm^−1^) 3003, 2928, 1722, 1671 (C=N), 1511, 1437, 1224, 696; ^1^H-NMR (CDCl_3_): δ (ppm) 3.30 (br. s., 2H, –C**H_2_**–CH_2_–N), 4.25 (br. s., 2H, –CH_2_–C**H_2_**–N), 5.26 (s, 2H, –C**H_2_**–C_6_H_4_), 6.92 (s, 1H, –N–C**H**=CH–N=), 7.08 (s, 1H, –N–CH=C**H**–N=), 7.44–7.52 (m, 9H, Ar–H), 7.55 (s, 1H, –N–CH=N–); ^13^C-NMR (CDCl_3_): δ (ppm) 29.2 (–**C**H_2_–CH_2_–N), 43.4 (–CH_2_–**C**H_2_–N), 75.7 (–**C**H_2_–C_6_H_4_), 118.8 (–N–**C**H=CH–N=), 125.9, 128.5, 128.6, 129.1, 129.6, 129.7, 133.8, 134.5, 135.9 (Ar–CH, Ar–C, –N–CH=**C**H–N=), 136.8 (–N–CH=N–), 155.0 (C=N); MS *m*/*z* (ESI): 340.1 [M + H]^+^, 341.1 [(M + 1) + H]^+^, 342.1 [(M + 2) + H]^+^.

*(1E)-N-[(4-Fluorobenzyl)oxy]-3-(1H-imidazol-1-yl)-1-phenylpropan-1-imine* (**Vd**). Yield 59.9%; light brown viscous oil; IR (KBr): ν (cm^−1^) 3003, 2924, 1720, 1670 (C=N), 1510, 1435, 1217, 765; ^1^H-NMR (CDCl_3_): δ (ppm) 3.29 (t, *J* = 6.6 Hz, 2H, –C**H_2_**–CH_2_–N), 4.25 (t, *J* = 6.5 Hz, 2H, –CH_2_–C**H_2_**–N), 5.27 (s, 2H, –C**H_2_**–C_6_H_4_), 6.90 (s, 1H, –N–C**H**=CH–N=), 7.08 (s, 1H, –N–CH=C**H**–N=), 7.15–7.22 (m, 2H, Ar–H), 7.45–7.56 (m, 8H, Ar–H, –N–CH=N–), ^13^C-NMR (CDCl_3_): δ (ppm) 29.2 (–**C**H_2_–CH_2_–N), 43.5 (–CH_2_–**C**H_2_–N), 75.9 (–**C**H_2_–C_6_H_4_), 115.3 (d, *J*_C–3′, F&C–5′, F_ = 21.1 Hz, C–3′ and C–5′), 118.9 (–N–**C**H=CH–N=), 125.9, 128.7, 128.8, 129.6, 134.6 (Ar–CH, Ar–C, –N–CH=**C**H–N=), 130.3 (d, *J*_C–2′, F&C–6′, F_ = 8.3 Hz, C–2′ and C–6′), 133.2 (d, *J*_C–1′, F_ = 2.8 Hz, C–1′), 136.8 (–N–CH=N–), 154.9 (C=N), 162.5 (d, *J*_C–4′, F_ = 246.9 Hz, C–4′); MS *m*/*z* (ESI): 324.2 [M + H]^+^, 325.2 [(M + 1) + H]^+^.

*(1E)-3-(1H-Imidazol-1-yl)-N-[(4-methylbenzyl)oxy]-1-phenylpropan-1-imine* (**Ve**). Yield 52.2%; light brown viscous oil; IR (KBr): ν (cm^−1^) 3003, 2781, 1714, 1676 (C=N), 1512, 1220, 790; ^1^H-NMR (CDCl_3_): δ (ppm) 2.41 (s, 3H, CH_3_), 3.25 (br. s., 2H, –C**H_2_**–CH_2_–N), 4.22 (br. s., 2H, –CH_2_–C**H_2_**–N), 5.26 (s, 1H, –C**H_2_**–C_6_H_4_), 6.95 (s, 1H, –N–CH=C**H**–N=), 7.04 (s, 1H, –N–C**H**=CH–N=), 7.26 (d, *J* = 6.3 Hz, 2H, Ar–H), 7.37 (d, *J* = 6.5 Hz, 2H, Ar–H), 7.41–7.51 (m, 5H, Ar–H), 7.64 (s, 1H, –N–CH=N–); ^13^C-NMR (CDCl_3_): δ (ppm) 21.2 (CH_3_), 29.4 (–**C**H_2_–CH_2_–N), 43.4 (–CH_2_–**C**H_2_–N), 76.6 (–**C**H_2_–C_6_H_4_), 118.9 (–N–**C**H=CH–N=), 125.9, 127.3, 128.5, 128.6, 129.1, 129.5, 129.6, 134.3, 134.9 (Ar–CH, Ar–C, –N–CH=**C**H–N=), 137.9 (–N–CH=N–), 154.7 (C=N); MS *m*/*z* (ESI): 320.2 [M + H]^+^, 321.2 [(M + 1) + H]^+^.

*(1E)-3-(1H-Imidazol-1-yl)-1-phenyl-N-{[4-(trifluoromethyl)benzyl]oxy}propan-1-imine* (**Vf**). Yield 43.3%; light brown viscous oil; IR (KBr): ν(cm^−1^) 3003, 2975, 1717, 1676 (C=N), 1502, 1437, 1219, 790; ^1^H-NMR (CDCl_3_): δ (ppm) 3.27 (br. s., 2H, –C**H_2_**–CH_2_–N), 4.21 (br. s., 2H, –CH_2_–C**H_2_**–N), 5.27 (s, 1H, –C**H_2_**–C_6_H_4_), 6.86 (s, 1H, –N–C**H**=CH–N=), 7.01 (s, 1H, –N–CH=C**H**–N=), 7.39–7.51 (m, 9H, Ar–H), 7.65 (s, 1H, –N–CH=N–); ^13^C-NMR (CDCl_3_): δ (ppm) 29.2 (–**C**H_2_–CH_2_–N), 43.6 (–CH_2_–**C**H_2_–N), 75.7 (–**C**H_2_–C_6_H_4_), 119.0 (–N–**C**H=CH–N=), 125.4, 125.5, 126.1, 127.4, 128.3, 128.8, 129.0, 129.8, 134.4, 136.9, 141.6 (Ar–CH, Ar–C, –N–CH=**C**H–N=, –N–CH=N–), 155.4 (C=N); MS *m*/*z* (ESI): 374.2 [M + H]^+^, 375.1 [(M + 1) + H]^+^.

*(1E)-N-{[3,5-Bis(trifluoromethyl)benzyl]oxy}-3-(1H-imidazol-1-yl)-1-phenylpropan-1-imine* (**Vg**). Yield 28%; light brown viscous oil; IR (KBr): ν (cm−^1^) 3003, 2970, 1717,1673 (C=N), 1502, 1420, 1223, 702; ^1^H-NMR (CDCl_3_): δ (ppm) 3.31 (br. s., 2H, –C**H_2_**–CH_2_–N), 4.25 (br. s., 2H, –CH_2_–C**H_2_**–N), 5.29 (s, 1H, –C**H_2_**–C_6_H_4_), 6.89 (s, 1H, –N–C**H**=CH–N=), 7.05 (s, 1H, –N–CH=C**H**–N=), 7.39–7.52 (m, 6H, Ar–H, –N–CH=N–), 7.75–7.86 (m, 2H, Ar–H), 8.39 (s, 1H, Ar–H); ^13^C-NMR (CDCl_3_): δ (ppm) 28.9 (–**C**H_2_–CH_2_–N), 44.3 (–CH_2_–**C**H_2_–N), 74.9 (–**C**H_2_–C_6_H_4_), 119.4 (–N–**C**H=CH–N=), 121.9, 122.5, 124.0, 126.2, 128.3, 128.9, 130.2, 131.7, 131.9, 136.6, 140.1 (Ar–CH, Ar–C, –N–CH=**C**H–N=, –N–CH=N–), 155.8 (C=N); MS *m*/*z* (ESI): 442.1 [M + H]^+^, 443.1 [(M + 1) + H]^+^.

*(1E)-1-(1,3-Benzodioxol-5-yl)-3-(1H-imidazol-1-yl)-N-(benzyloxy)propan-1-imine* (**Vh**). Yield 61%; light brownviscousoil; IR (KBr): ν (cm^−1^) 3030, 2927, 1670 (C=N), 1606, 1489, 1284, 700; ^1^H-NMR (CDCl_3_): δ (ppm) 3.17 (t, *J* = 7.0 Hz, 2H, –C**H_2_**–CH_2_–N), 4.18 (t, *J* = 7.0 Hz, 2H, –CH_2_–C**H_2_**–N), 5.22 (s, 2H, –C**H_2_**–C_6_H_5_), 5.99 (s, 2H, –O–CH_2_–O–), 6.76 (d, *J* = 8.0 Hz, 1H, Ar–H), 6.82 (s, 1H, –N–C**H**=CH–N=), 6.86 (dd, *J* = 1.5, 8.5 Hz, 1H, Ar–H), 7.01 (s, 1H, –N–CH=C**H**–N=), 7.09 (d, *J* = 1.0 Hz, 1H, Ar–H), 7.33–7.37 (m, 5H, Ar–H), 7.50 (s, 1H, –N–CH=N–); ^13^C-NMR (CDCl_3_): δ (ppm) 29.4 (–**C**H_2_–CH_2_–N), 43.7 (–CH_2_–**C**H_2_–N), 76.2 (–**C**H_2_–C_6_H_4_), 101.4 (–O–CH_2_–O–), 106.2, 108.2 (Ar–CH), 119.0 (–N–**C***H*=CH–N=), 120.3, 128.2, 128.4, 128.5,128.7, 129.0, 136.9 (Ar–CH, Ar–C, –N–CH=**C**H–N=), 137.5 (–N–CH=N–), 148.2, 148.3 (Ar–C), 154.3 (C=N); MS *m*/*z* (ESI): 350.1 [M + H]^+^.

*(1E)-1-(1,3-Benzodioxol-5-yl)-3-(1H-imidazol-1-yl)-N-[(4-bromobenzyl)oxy]propan-1-imine* (**Vi**). Yield 55%; pale yellow solid, m.p. 80–82 °C; IR (KBr): ν (cm^−1^) 3115, 2934, 1670 (C=N), 1506, 1489, 1232, 756; ^1^H-NMR (CDCl_3_): δ (ppm) 3.17 (t, *J* = 7.0 Hz, 2H, –C**H_2_**–CH_2_–N), 4.17 (t, *J* = 7.0 Hz, 2H, –CH_2_–C**H_2_**–N), 5.13 (s, 2H, –C**H_2_**–C_6_H_4_), 5.98 (s, 2H, –O–CH_2_–O–), 6.76 (d, *J* = 8.0 Hz, 1H, Ar–H), 6.84 (s, 1H, –N–C**H**=CH–N=), 6.88 (dd, *J* = 1.5, 8.0 Hz, 1H, Ar–H), 7.01 (s, 1H, –N–CH=C**H**–N=), 7.07 (d, *J* = 1.0 Hz, 1H, Ar–H), 7.25 (d, *J* = 8.0 Hz, 2H, Ar–H), 7.50 (d, *J* = 8.5 Hz, 2H, Ar–H), 7.52 (s, 1H, –N–CH=N–); ^13^C-NMR (CDCl_3_): δ (ppm) 29.2 (–**C**H_2_–CH_2_–N), 43.7 (–CH_2_–**C**H_2_–N), 75.8 (–**C**H_2_–C_6_H_4_), 101.5 (–O–CH_2_–O–), 106.2, 108.2 (Ar–CH), 119.0 (–N–**C**H=CH–N=), 120.4, 122.1, 128.7, 128.9, 130.1, 131.5, 136.5 (Ar–CH, Ar–C, –N–CH=**C**H–N=), 136.9 (–N–CH=N–), 148.2, 149.0 (Ar–C), 154.6 (C=N); MS *m*/*z* (ESI): 428.1 [M + H]^+^, 430.0 [(M + 2) + H]^+^, 431.0[(M + 3) + H]^+^.

*(1E)-1-(1,3-Benzodioxol-5-yl)-3-(1H-imidazol-1-yl)-N-[(4-chlorobenzyl)oxy]propan-1-imine* (**Vj**). Yield 40%; light brown viscous oil; IR (KBr): ν (cm^−1^) 3017, 2932, 1670 (C=N), 1506, 1491, 1280, 756; ^1^H-NMR (CDCl_3_): δ (ppm) 3.16 (t, *J* = 7.0 Hz, 2H, –C**H_2_**–CH_2_–N), 4.15 (t, *J* = 7.0 Hz, 2H, –CH_2_–C**H_2_**–N), 5.15 (s, 2H, –C**H_2_**–C_6_H_4_), 5.98 (s, 2H, –O–CH_2_–O–), 6.76 (d, *J* = 8.0 Hz, 1H, Ar–H), 6.84 (s, 1H, –N–C**H**=CH–N=), 6.86 (dd, *J* = 1.5, 8.0 Hz, 1H, Ar–H), 7.01 (s, 1H, –N–CH=C**H**–N=), 7.07 (d, *J* = 1.0 Hz, 1H, Ar–H), 7.31–7.33 (m, 2H, Ar–H), 7.35 (d, *J* = 8.5 Hz, 2H, Ar–H), 7.43 (s, 1H, –N–CH=N–); ^13^C-NMR (CDCl_3_): δ (ppm) 29.3 (–**C**H_2_–CH_2_–N), 43.6 (–CH_2_–**C**H_2_–N), 76.7 (–**C**H_2_–C_6_H_4_), 101.5 (–O–CH_2_–O–), 106.2, 108.2 (Ar–CH), 118.9 (–N–**C**H=CH–N=), 120.4, 121.9, 128.7, 129.3, 129.7, 133.9, 136.5 (Ar–CH, Ar–C, –N–CH=**C**H–N=), 136.9 (–N–CH=N–), 148.2, 149.0 (Ar–C), 154.6 (C=N); MS *m*/*z* (ESI): 384.1 [M + H]^+^, 385.1 [(M + 1) + H]^+^, 386.1 [(M + 2) + H]^+^.

*(1E)-1-(1,3-Benzodioxol-5-yl)-3-(1H-imidazol-1-yl)-N-[(4-fluorobenzyl)oxy]propan-1-imine* (**Vk**). Yield 32%; light brown viscous oil; IR (KBr): ν (cm^−1^) 3018, 2962, 1604 (C=N), 1510, 1490, 1215, 759; ^1^H-NMR (CDCl_3_): δ (ppm) 3.14 (t, *J* = 7.1 Hz, 2H, –C**H_2_**–CH_2_–N), 4.14 (t, *J* = 7.0 Hz, 2H, –CH_2_–C**H_2_**–N), 5.14 (s, 2H, –C**H_2_**–C_6_H_4_), 5.96 (s, 2H, –O–CH_2_–O–), 6.75 (d, *J* = 8.1 Hz, 1H, Ar–H), 6.82 (s, 1H, –N–C**H**=CH–N=), 6.86 (dd, *J* = 2.0, 8.5 Hz, 1H, Ar–H), 6.99 (s, 1H, –N–CH=C**H**–N=), 7.04–7.07 (m, 3H, Ar–H), 7.35–7.38 (m, 2H, Ar–H), 7.41 (s, 1H, –N–CH=N–); ^13^C-NMR (CDCl_3_): δ (ppm) 29.3 (–**C**H_2_–CH_2_–N), 43.6 (–CH_2_–**C**H_2_–N), 75.9 (–**C**H_2_–C_6_H_4_), 101.5 (–O–CH_2_–O–), 106.2, 108.2 (Ar–CH), 115.4 (d, *J*_C–3′, F&C–5′, F_= 21.4 Hz, C–3′ and C–5′), 119.0 (–N–**C**H=CH–N=), 120.3, 128.9, 129.3, (Ar–CH, Ar–C, –N–CH=**C**H–N=), 130.3 (d, *J*_C–2′, F&C–6′, F_= 8.2 Hz, C–2′ and C–6′), 133.3 (d, *J*_C–1′, F_ = 3.2 Hz, C–1′), 136.9 (–N–CH=N–), 148.2, 149.0 (Ar–C), 154.5 (C=N), 162.6 (d, *J*_C–4′, F_= 246.4 Hz, C–4′); MS *m*/*z* (ESI): 368.1 [M + H]^+^, 369.1 [(M + 1) + H]^+^.

*(1E)-1-(1,3-Benzodioxol-5-yl)-3-(1H-imidazol-1-yl)-N-[(4-methylbenzyl)oxy]propan-1-imine* (**Vl**). Yield 24%; light brown viscous oil; IR (KBr): ν (cm^−1^) 3669, 3115, 2953, 1614 (C=N), 1585, 1510, 1248, 754; ^1^H-NMR (CDCl_3_): δ (ppm) 2.39 (s, 3H, CH_3_), 3.15 (t, *J* = 7.0 Hz, 2H, –C**H_2_**–CH_2_–N), 4.17 (t, *J* = 7.0 Hz, 2H, –CH_2_–C**H_2_**–N), 5.18 (s, 2H, –C**H_2_**–C_6_H_4_), 5.99 (s, 2H, –O–CH_2_–O–), 6.77 (d, *J* = 8.0 Hz, 1H, Ar–H), 6.83 (s, 1H, –N–C**H**=CH–N=), 6.88 (dd, *J* = 1.5, 8.0 Hz, 1H, Ar–H), 7.01 (s, 1H, –N–CH=C**H**–N=), 7.09 (d, *J* = 1.5 Hz, 1H, Ar–H), 7.22 (d, *J* = 7.5 Hz, 2H, Ar–H), 7.32 (d, *J* = 8.0 Hz, 2H, Ar–H), 7.46 (s, 1H, –N–CH=N–); ^13^C-NMR (CDCl_3_): δ (ppm) 21.2 (*C*H_3_), 29.4 (–**C**H_2_–CH_2_–N), 43.7 (–CH_2_–**C**H_2_–N), 76.6 (–**C**H_2_–C_6_H_4_), 101.4 (–O–CH_2_–O–), 106.2, 108.2 (Ar–CH), 118.9 (–N–**C**H=CH–N=), 120.4, 128.6, 128.9, 129.1, 129.2, 134.4, 136.9 (Ar–CH, Ar–C, –N–CH=**C**H–N=), 137.9 (–N–CH=N–), 148.1, 148.9 (Ar–C), 154.2 (C=N); MS *m*/*z* (ESI): 364.1 [M + H]^+^.

*(1E)-1-(1,3-Benzodioxol-5-yl)-3-(1H-imidazol-1-yl)-N-{[4-(trifluromethyl)benzyl]oxy}propan-1-imine* (**Vm**). Yield 25%; light brown solid, m.p. 81–83 °C; IR (KBr): ν (cm^−1^) 3016, 2941, 1670 (C=N), 1506, 1448, 1232, 756; ^1^H-NMR (CDCl_3_): δ (ppm) 3.23 (t, *J* = 7.0 Hz, 2H, –C**H_2_**–CH_2_–N), 4.23 (t, *J* = 7.0 Hz, 2H, –CH_2_–C**H_2_**–N), 5.24 (s, 2H, –C**H_2_**–C_6_H_4_), 5.99 (s, 2H, –O–CH_2_–O–), 6.78 (d, *J* = 8.1 Hz, 1H, Ar–H), 6.86 (s, 1H, –N–C**H**=CH–N=), 6.91 (dd, *J* = 1.6, 8.1 Hz, 1H, Ar–H), 7.04 (s, 1H, –N–CH=C**H**–N=), 7.09 (d, *J* = 1.4 Hz, 1H, Ar–H), 7.49 (d, *J* = 7.7 Hz, 2H, Ar–H), 7.65 (d, *J* = 7.9 Hz, 2H, Ar–H), 7.68 (s, 1H, –N–CH=N–); ^13^C-NMR (CDCl_3_): δ (ppm) 29.1 (–**C**H_2_–CH_2_–N), 43.9 (–CH_2_–**C**H_2_–N), 75.6 (–**C**H_2_–C_6_H_4_), 101.5 (–O–CH_2_–O–), 106.2, 108.3 (Ar–CH), 119.1 (–N–**C**H=CH–N=), 120.5, 123.0, 125.4, 125.5, 128.4, 128.5, 130.1, 136.8, 141.6 (Ar–CH, Ar–C, –N–CH=**C**H–N=, –N–CH=N–), 148.3, 149.2 (Ar–C), 154.8 (C=N); MS *m*/*z* (ESI): 418.1 [M + H]^+^, 419.1 [(M + 1) + H]^+^.

*(1E)-1-(1,3-Benzodioxol-5-yl)-3-(1H-imidazol-1-yl)-N-{[3,5-bis(trifluromethyl)benzyl]oxy} propan-1-imine* (**Vn**). Yield 55%; light brown viscous oil; IR (KBr): ν (cm−1) 3014, 2900, 1670 (C=N), 1504, 1446, 1232, 754; ^1^H-NMR (CDCl_3_): δ (ppm) 3.24 (t, *J* = 6.9 Hz, 2H, –C**H_2_**–CH_2_–N), 4.22 (t, *J* = 6.9 Hz, 2H, –CH_2_–C**H_2_**–N), 5.26 (s, 2H, –C**H_2_**–C_6_H_4_), 5.99 (s, 2H, –O–CH_2_–O–), 6.78 (d, *J* = 8.1 Hz, 1H, Ar–H), 6.89 (s, 1H, –N–C**H**=CH–N=), 6.92 (dd, *J* = 1.5, 8.1 Hz, 1H, Ar–H), 7.03 (s, 1H, –N–CH=C**H**–N=), 7.06 (d, *J* = 1.3 Hz, 1H, Ar–H), 7.65 (s, 1H, –N–CH=N–), 7.84–7.86 (m, 3H, Ar–H); ^13^C-NMR (CDCl_3_): δ (ppm) 29.1 (–**C**H_2_–CH_2_–N), 43.9 (–CH_2_–**C**H_2_–N), 74.8 (–**C**H_2_–C_6_H_4_), 101.6 (–O–CH_2_–O–), 106.2, 108.3 (Ar–CH), 119.1 (–N–**C**H=CH–N=), 120.6, 121.9, 124.4, 128.1, 128.2, 131.7, 131.9, 136.8, 140.3 (Ar–CH, Ar–C, –N–CH=**C**H–N=, –N–CH=N–), 148.3, 149.4 (Ar–C), 155.4 (C=N); MS *m*/*z* (ESI): 486.1 [M + H]^+^, 487.1 [(M + 1) + H]^+^.

### 3.3. Crystal Structure Determination

Compound **Vi** was obtained as single crystals by slow evaporation from ethanolic solution of the pure compound at room temperature. Data were collected on a Bruker APEX-II D8 Venture area diffractometer, equipped with graphite monochromatic Mo Kα radiation, λ = 0.71073 Å at 293 (2) K. Cell refinement and data reduction were carried out by Bruker SAINT. SHELXT [22,23] was used to solve the structure. The final refinement was performed by full-matrix least-squares techniques with anisotropic thermal data for non-hydrogen atoms. In compound **Vi**, C_20_H_18_BrN_3_O_3,_ the crystallographic data and refinement information are summarized in Table S1. The crystallographic data of compound **Vi** have been deposited with the Cambridge Crystallographic Data Center (CCDC-1577844) and can be found in Supplementary Materials. Copies of the data may be obtained free of charge from the Director, CCDC, 12 Union Road, Cambridge, CB2 1EZ, UK (deposit@ccdc.cam.ac.uk).

### 3.4. Antifungal Activity

#### 3.4.1. Materials

The reference standard antifungal drugs, fluconazole and ketoconazole, were obtained from Shouguang-Fukang Pharmaceutical Ltd. (Weifang, China) and from Sigma-Aldrich Co. (St. Louis, MO, USA), respectively. Liquid RPMI 1640 medium supplemented with l-glutamine was purchased from Gibco-BRL, Life Technologies (Paisley, Scotland). Sabouraud Dextrose Agar (SDA) was obtained from Merck Co. (Darmstadt, Germany). Dimethyl sulfoxide (100%) was used to dissolve the reference standards and/or the tested compounds **Va**–**n** to afford an initial concentration of 2048 mg/L.

#### 3.4.2. Organisms

*Candida albicans* (ATCC 90028), *Candida tropicalis* (ATCC 66029), *Candida parapsilosis* (ATCC 22019), and *Aspergillus niger* (ATCC 16404) were used to assess antifungal activity.

#### 3.4.3. Preparation of Fungal Inocula

Fungal inocula were prepared as previously reported [21].

#### 3.4.4. Preparation of the Tested Compound Solutions

Briefly, a twofold dilution series of the tested compounds **Va**–**n** was prepared in a double-strength RPMI 1640 culture medium. Ten serial dilutions were prepared to afford concentrations ranging from 1024 mg/L to 2 mg/L.

#### 3.4.5. Antifungal Susceptibility Studies

The MIC values of the tested compounds **Va**–**n** were determined as previously reported [21].

## 4. Conclusions

The synthesis and spectroscopic characterization of certain new oximino ethers **Va**–**n** bearing imidazole pharmacophore moiety have been reported. Single crystal X-ray analysis of compound **Vi** confirmed the assigned (*E*)-configuration of the imine functionality of the target compounds **Va**–**n**. The in vitro antifungal potential of compounds **Va**–**n** was assessed using DIZ and MIC assays. Compound **Ve** emerged as the most active compound toward *Candida albicans*, being nearly equipotent with the reference antifungal drug FLC with an MIC value of 0.050 µmol/mL. On the other hand, compounds **Vi** and **Vn** exhibited the most potent activity towards *Candida parapsilosis*, with an MIC value of 0.002 µmol/mL—about twenty-three times more potent than FLC. It seems that the replacement of the phenyl ring in compounds **Va**–**g** with the 1,3-benzodioxole scaffold, which gave their respective compounds **Vh**–**n**, did not show superior antifungal activity against the tested fungal strains except towards *Candida parapsilosis.* The antifungal results of the current investigation might support the development of new potent and safer azole antifungal agents to be harnessed in the clinic.

## Figures and Tables

**Table 1:** Selected geometric parameters (Å, °).

**Bond Lengths**			
Br1–C18	1.905(3)	N1–C8	1.290(4)
O1–C1	1.432(5)	N2–C10	1.462(5)
O1–C2	1.386(4)	N2–C11	1.337(5)
O2–C1	1.428(5)	N2–C13	1.347(6)
O2–C7	1.373(4)	N3–C11	1.313(6)
O3–N1	1.410(4)	N3–C12	1.343(6)
O3–C14	1.442(4)		
**Bond Angles**			
C1–O1–C2	105.9(3)	O2–C7–C2	110.5(3)
C1–O2–C7	105.9(3)	O2–C7–C6	128.4(3)
N1–O3–C14	107.6(2)	N1–C8–C4	114.9(3)
O3–N1–C8	111.8(3)	N1–C8–C9	123.1(3)
C10–N2–C11	127.2(3)	N2–C10–C9	112.3(3)
C10–N2–C13	126.8(3)	N2–C11–N3	112.3(4)
C11–N2–C13	106.0(3)	N3–C12–C13	110.2(4)
C11–N3–C12	104.8(4)	N2–C13–C12	106.7(4)
O1–C1–O2	108.0(3)	O3–C14–C15	113.3(3)
O1–C2–C3	127.9(3)	Br1–C18–C17	119.4(2)
O1–C2–C7	109.3(3)	Br1–C18–C19	119.3(3)

**Table 2:** Hydrogen-bond geometry (Å, °).

D–H···A	D–H	H···A	D···A	D–H···A
C3–H3A···N3	0.9300	2.5300	3.453(5)	169.00
Symmetry codes: (i) –*x* − 2, −*y*, −*z* + 1.				

**Table 3:** Antifungal activity of the target oximino ethers **Va**–**n** against *C*. *albicans*, *C. tropicalis*, *C. parapsilosis* and *A. niger.*

Compound No.	*Candida albicans*		*Candida tropicalis*		*Candida parapsilosis*		*Asperagillus niger*	
	DIZ ± SD (mm)	MIC (µmol/mL)	DIZ ± SD (mm)	MIC (µmol/mL)	DIZ ± SD (mm)	MIC (µmol/mL)	DIZ ± SD (mm)	MIC (µmol/mL)
**Va**	18 ± 1.00	0.209	16 ± 1.16	0.419	20 ± 0.68	0.105	21 ± 1.14	0.105
**Vb**	23 ± 0.63	0.083	18 ± 0.97	0.083	24 ± 0.45	0.042	22 ± 0.94	0.083
**Vc**	22 ± 0.52	0.094	22 ± 0.63	0.094	23 ± 0.61	0.047	19 ± 0.75	0.047
**Vd**	18 ± 1.12	0.198	15 ± 1.21	0.198	19 ± 0.85	0.049	12 ± 0.30	0.198
**Ve**	19 ± 1.10	0.050	17 ± 1.14	0.100	20 ± 1.00	0.050	12 ± 0.41	0.201
**Vf**	18 ± 0.95	0.686	18 ± 1.13	0.343	21 ± 0.43	0.043	22 ± 0.99	0.172
**Vg**	21 ± 1.13	0.073	20 ± 0.91	0.145	22 ± 0.58	0.036	13 ± 0.64	0.290
**Vh**	14 ± 0.50	0.183	10 ± 0.58	0.183	13 ± 0.60	0.003	20 ± 0.50	0.733
**Vi**	15 ± 1.20	0.149	15 ± 0.30	0.149	14 ± 0.50	0.002	13 ± 1.53	0.299
**Vj**	15 ± 0.30	0.167	14 ± 0.60	0.167	21 ± 1.00	0.010	16 ± 1.31	0.667
**Vk**	15 ± 0.58	0.174	12 ± 0.58	0.174	13 ± 0.58	0.044	23 ± 0.50	0.697
**Vl**	16 ± 1.00	0.176	13 ± 0.60	0.176	19 ± 1.00	0.006	12 ± 0.58	0.705
**Vm**	15 ± 0.58	0.153	13 ± 0.40	0.153	23 ± 0.60	0.019	14 ± 1.00	0.307
**Vn**	13 ± 0.40	0.527	15 ± 1.20	0.527	19 ± 0.58	0.002	11 ± 0.20	>1.05
Fluconazole	18 ± 1.10	0.051	19 ± 1.00	0.045	19 ± 0.90	0.047	ND	ND
Ketokonazole	ND	ND	ND	ND	ND	ND	29 ± 0.60	0.02

Arithmetic mean ± standard deviation; DIZ: diameter of the inhibition zone; SD: standard deviation; MIC: minimum inhibitory concentration; ND: not determined.

## Supplementary Materials

Supplementary Materials are available online.
