# Supplementary material for: Synthesis, Single Crystal X-ray Analysis, and Antifungal Profiling of Certain New Oximino Ethers Bearing Imidazole Nuclei
Source: Molecules. 2017 Nov 3;22(11):1895. doi: 10.3390/molecules22111895 (PMC6150221; doi:10.3390/molecules22111895)
Supplement: Supplementary file 1 [file molecules-22-01895-s001.zip › Supplementary.pdf]

Synthesis, single crystal X-ray analysis and antifungal profiling of certain new oximino ethers bearing imidazole nuclei

Reem I. Al-Wabli[1]*, Alwah R. Al-Ghamdi[1], Hazem A. Ghabbour[1,2], Mohamed H. Al-Agamy[3,4] and Mohamed I. Attia[1,5]*

[1]Department of Pharmaceutical Chemistry, College of Pharmacy, King Saud University, P.O. Box 2457, Riyadh 11451, Saudi Arabia
[2]Department of Medicinal Chemistry, Faculty of Pharmacy, Mansoura University, Mansoura 35516, Egypt
[3]Department of Pharmaceutics, College of Pharmacy, King Saud University, P.O. Box 2457, Riyadh 11451,
Saudi Arabia; malagamy@ksu.edu.sa
[4]Microbiology and Immunology Department, Faculty of Pharmacy, Al-Azhar University, Cairo 11884, Egypt
[5]Medicinal and Pharmaceutical Chemistry Department, Pharmaceutical and Drug Industries Research Division, National Research Centre (ID: 60014618), El Bohooth Street, Dokki, Giza 12622, Egypt

Table S1. The X-ray experimental details of compound **Vi**.

**Crystal data**	
Chemical formula	$C_{20}H_{18}BrN_3O_3$
Mr	428.28
Crystal system, space group	Monoclinic, $P2_1/c$
Temperature (K)	293
a, *b*, *c* (Å)	18.7879 (14), 5.8944 (4), 16.7621 (12)
β (°)	91.632 (2)
V (Å3)	1855.5 (2)
Z	4
Radiation type	Mo *Kα*
μ (mm^{-1})	2.24
Crystal size (mm)	0.33 × 0.28 × 0.08
**Data collection**	
Diffractometer	Bruker APEX-II D8 venture diffractometer
Absorption correction	Multi-scan,SADABS Bruker 2014
Tmin, Tmax	0.528, 0.846
No. of measured, independent and observed [I> 2σ(I)] reflections	35254, 3848, 2376
R_{int}	0.136
**Refinement**	
R[F^2> 2σ(F^2)], wR(F^2), S	0.048, 0.111, 1.02
No. of reflections	3848
No. of parameters	244
No. of restraints	0
H-atom treatment	H atoms treated by a mixture of independent and constrained refinement
$\Delta\rho_{max}$, $\Delta\rho_{min}$ (e $Å^{-3}$)	0.37, −0.43
